# Endocrine consequences of childhood obesity: a narrative review

**DOI:** 10.3389/fendo.2025.1584861

**Published:** 2025-04-30

**Authors:** Maroun Badr, Ghazwa El-Rabaa, Marianne Freiha, Andrzej Kędzia, Elżbieta Niechciał

**Affiliations:** Department of Pediatric Diabetes, Clinical Auxology and Obesity, Poznan University of Medical Sciences, Poznan, Poland

**Keywords:** children, obesity, childhood obesity, obesity-related endocrinopathies, endocrine disorders, hormones

## Abstract

Childhood obesity has emerged as a significant public health challenge, with profound consequences that negatively impact endocrine functions. Excess adiposity in children leads to dysregulation of various hormonal pathways, notably insulin resistance and hyperinsulinemia, the best-established endocrine changes in obesity. If insulin resistance is not adequately managed, it might precipitate type 2 diabetes. Another common finding among children with obesity is thyroid dysfunction. Some studies suggest that obesity may be associated with alterations in thyroid hormone levels, potentially leading to hypothyroidism, although the relationship is complex and not fully understood. Additionally, obesity affects the hypothalamic-pituitary-gonadal axis, resulting in precocious puberty, particularly in girls. Elevated leptin levels, a hormone produced by adipose tissue, can contribute to a paradoxical state of leptin resistance, further complicating metabolic processes and appetite regulation. Moreover, childhood obesity can result in increased secretion of cortisol, which may enhance the risk of developing metabolic syndrome and cardiovascular complications. The interplay between obesity and endocrine function also extends to growth patterns, where excess weight can lead to growth acceleration followed by potential short stature in adulthood due to early epiphyseal closure. Addressing the endocrine consequences of childhood obesity requires a comprehensive approach that includes prevention, early intervention, and management strategies tailored to this vulnerable population. Understanding these complex interactions is crucial for developing effective public health policies to mitigate the impact of obesity on endocrine health in children. By reviewing research, this work provides a comprehensive overview of the most relevant endocrine consequences of childhood obesity.

## Introduction

Obesity is a chronic, recurrent disease defined by excessive fat deposits that can negatively impair health (1). It results from the intricate interplay of food consumption patterns, lifestyle behaviors, genetics, and socioeconomic factors (2). The prevalence of childhood obesity has increased significantly, reaching epidemic proportions in many countries. According to the World Health Organization (WHO), in 2022, around 37 million children under five years old were overweight, while in older children, the number of individuals aged 5–19 being overweight or obese achieved over 340 million globally, with both sexes holding approximately similar figures. The prevalence of obesity among children and adolescents aged 5–19 has risen significantly from just 2% in 1990 to 8% in 2022 (1). Previously, it was considered a high-income country health issue; nowadays, overweight and obesity are increasing in low- and middle-income countries (3).

The causes of childhood obesity are highly complex, involving genetic, environmental, behavioral, and socioeconomic factors. The primary cause of excessive body weight is an imbalance between calorie intake and output. However, the emergence of family forms of obesity reflects the role of genetic predisposition in the development of childhood obesity, with an inheritance rate of 25% (4). Although polygenic obesity is the most commonly observed, several single-gene defects and obesity-related syndromes have been identified (5). Syndromic childhood obesity includes around 25 rare forms, where obesity presents as part of a distinct set of clinical phenotypes, with Prader-Willi syndrome being the most common (5). Conversely, mutations in a single gene regulating body weight mainly through the leptin-melanocortin pathway and involving less than 1% of children in tertiary pediatric clinics reflect non-syndromic monogenic obesity (4, 5).

Specific genes increase the risk of obesity only when influenced by environmental and behavioral factors (4, 6). The modern change in lifestyle, including poor diet quality featuring high-calorie, low-nutrient foods and a decrease in physical activity, have notably played a significant part in the increased incidence of obesity (4–6). Sedentary lifestyles and poor sleep patterns are strongly associated with obesity, affecting dietary habits and hormone levels (5, 6).

In addition to that, several medical conditions contribute to the development of obesity, including endocrine disorders and damage to the central nervous system (6). These include endogenous or exogenous glucocorticoid excess and hypothyroidism, among many others (5). Medications such as steroids and antipsychotics are also associated with significant weight gain, necessitating proactive weight management strategies (4, 5).

Social factors, including low parental education, family stress, and unusual environments, also affect children’s eating behavior (4). These factors lead to maladaptive coping strategies, such as eating to suppress negative emotions, appetite up-regulation, and low-grade inflammation (4, 5).

This rise in obesity rates among children is alarming due to the associated health risks, including a long-term public health issue and a range of endocrine disorders. Childhood obesity has been significantly associated with metabolic complications that compromise what is known as metabolic syndrome (7). These complications include hypertension, and dyslipidemia, which are known to increase the risk of cardiovascular disease and type 2 diabetes (T2D) (7).

Moreover, recent evidence shows that there is a link between obesity and numerous endocrinological disturbances. Most endocrine changes are believed to occur secondary to the accumulation of extra fat mass because they may be induced by the enhanced production of free fatty acids, many peptides, and other adipokines by hypertrophic adipocytes (8).

This review provides a comprehensive overview of the endocrine consequences of childhood obesity.

## Methods

This review is narrative, and no systematic literature search was performed; each author identified and critically reviewed the most relevant papers. The work presents several studies on endocrine consequences of childhood obesity. The following electronic databases were searched: PubMed, Scopus, EMBASE, and Web of Science. The search time was up to February 2025 using the following keywords: childhood obesity; obesity; children; adolescents; endocrinology; hormones; endocrine changes; pituitary; thyroid; gonads; adrenals; pancreas; insulin sensitivity; insulin resistance; gastrointestinal hormones, adipose tissue. All articles published between January 2000 and February 2025 were checked by title, abstract, and full text. The following criteria for inclusion were applied in our search: relevant full-text articles in the English language, research articles, reviews, meta-analyses, and clinical studies discussing endocrine changes associated with obesity, including but not limited to insulin resistance, leptin, ghrelin, cortisol, and sex hormones. While exclusions criteria included: research primarily addressing non-endocrine health outcomes (e.g., cardiovascular disease, mental health issues) without a significant emphasis on endocrine consequences, articles not published in peer-reviewed journals, including conference abstracts or dissertations, studies published in languages other than the English language. It aimed to detect the most clinically significant papers related to the topic and provide a theoretical point of view, which is considered a valuable educational tool in continuing medical education.

### Thyroid gland

Obesity often alters thyroid function and structure, exhibiting similarities with Hashimoto thyroiditis but typically without the presence of autoantibodies (9). It has been shown that subclinical hypothyroidism, which is significantly more prevalent in children with obesity (14%) compared to healthy individuals (6.8%) (10), is a consequence rather than a causative factor of obesity (9). These findings seem to align with the results of studies showing higher levels of TSH in children with obesity, while T4 can be elevated (11) or unchanged (9), and T3 slightly increased (10). Despite these changes, thyroid hormone medication is not indicated if there is no evidence of autoimmune thyroid diseases or iodine deficiency. A study conducted by Licenziati et al. showed that weight loss tends to normalize the changes in thyroid function and structure caused by obesity (9).

**Figure 1:**
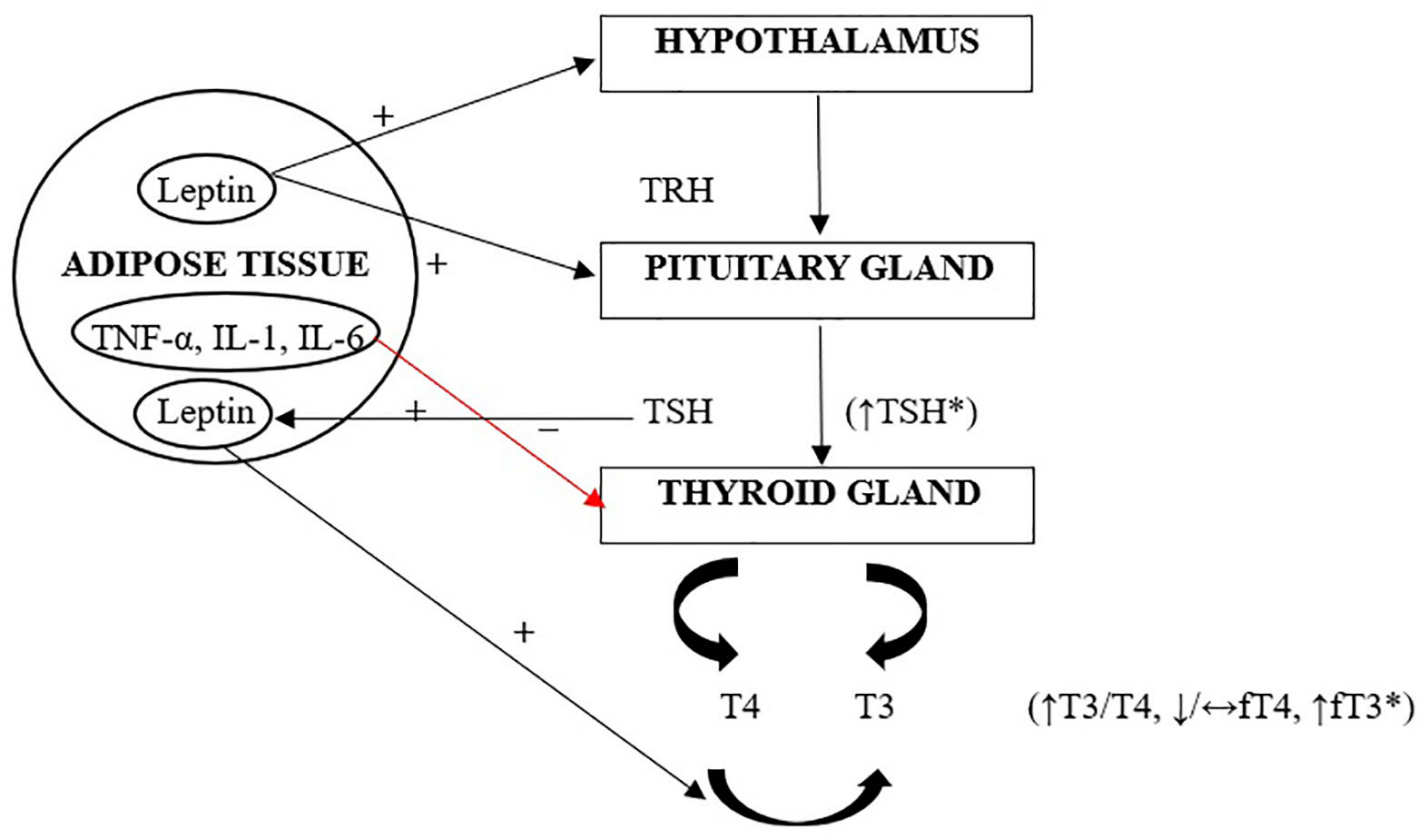
Thyroid axis in childhood obesity (10). Leptin, produced by adipose tissue, acts directly on the hypothalamic arcuate nucleus, thereby increasing the synthesis and secretion of TRH, and subsequently TSH. Increased secretion of TSH, as well as elevated levels of fT3, are considered defensive mechanisms against further weight gain. Additionally, TSH directly influences the differentiation of preadipocytes into adipocytes, which consequently increases leptin levels. Leptin-stimulated increased activity of type 1 and type 2 deiodinases (D1 and D2) leads to an increase in the tissue pool of T3. Cytokines secreted by adipose tissue that promote inflammation affect the reduction of sodium-iodide symporter expression in the thyroid, which may potentially inhibit iodine uptake by the gland, resulting in a compensatory increase in TSH. TRH, thyrotropin-releasing hormone; TSH, thyroid stimulating hormone; T3, Triiodothyronine; T4, thyroxine; fT4, free thyroxine; FT3, free triiodothyronine; TNF-α, tumor necrosis factor; IL-1, interleukin-1; IL-6, interleukin-6.

Several hypotheses could explain these changes in the thyroid gland. Firstly, leptin, which is released by adipocytes, with a serum level correlating with body fat, conveys fat storage to the central nervous system, controlling hunger and energy expenditure (12). On the thyroid level, leptin acts on the hypothalamic-pituitary-thyroid axis, stimulating the release of thyrotropin-releasing hormone (TRH) and increasing TSH and thyroid hormone levels. Secondly, the elevated levels of TSH and T3 may also be explained by thyroid hormone resistance caused by a decrease in TSH or peripheral thyroid hormone receptors (10). Furthermore, obesity is often associated with increased inflammatory cytokines, which have been shown to impair thyroid activity and limit iodide uptake, raising TSH levels (9). Lastly, these alterations could result from an adaptation mechanism that increases energy expenditure, preventing further weight gain (10). **Figure 1** gives an overview of thyroid axis function in youth with obesity.

Ultrasounds of the thyroid in obese individuals often reveal a hypoechoic pattern. These structural changes usually improve after weight loss and are generally attributed to the low-grade inflammation seen in obesity (9). Insulin resistance, commonly associated with obesity, also affects the thyroid. It has been associated with the development of nodules and an increased risk of thyroid cancer, further confirming the link between obesity and thyroid irregularities (13).

### Adrenal glands

Obesity is often associated with an increased responsiveness of the hypothalamic-pituitary-adrenal (HPA) axis, increasing cortisol secretion. Despite this hyperactivity, most individuals will have a low or normal plasma cortisol and an elevated urinary free-cortisol level. The cortisol concentration in the plasma is carefully balanced between synthesis and clearance. Therefore, the low or normal plasma cortisol level seen in obese individuals may be explained by increased clearance (14). This leads to decreased negative feedback at the hypothalamus and pituitary, increasing HPA stimulation.

In addition to being centrally regulated, cortisol availability in peripheral tissue relies on 11β-hydroxysteroid dehydrogenase (11β-HSD) activity. At least two isozymes catalyze the inter-conversion of hormonally active (cortisol) and inactive (cortisone) metabolites. By converting cortisol to cortisone, 11β-HSD2, which is mainly expressed in the kidneys, protects the mineralocorticoid receptor from glucocorticoid excess. Insulin target tissues like the liver, muscle, and adipose tissue express the other isoform, 11β-HSD1 (15). An increased expression of 11β-HSD1 is observed in adipose stromal cells from omental fat, stimulating the local cortisol secretion via the conversion of cortisone to cortisol. The rise in local cortisol will activate the glucocorticoid receptor and further promote central adiposity (16). In their study, Masuzaki et al. found an increase in the adipose levels of corticosterone in the 11β-HSD1 transgenic mice, which led to an increase in visceral adipocytes size, possibly due to the higher glucocorticoid receptor at this site (17).

The constant over-activation of the renin-angiotensin-aldosterone (RAAS) system, both systemically and in adipocytes, increasing aldosterone secretion, is another essential condition to consider (18).

This contributes to hypertension (18), insulin resistance (19), and a heightened risk of cardiovascular diseases (20).

In addition to the activation of RAAS, various mechanisms may contribute to the increase of aldosterone and renin in obesity, such as activating the sympathetic nervous system (18), excessive secretion of adipocyte-derived cytokines, and an increase in the mineralocorticoid receptor in adipocytes (21).

An increase in aldosterone levels in obese individuals was noted irrespective of ACTH level, potassium, or renal function (19). Weight loss has decreased renin and aldosterone levels, highlighting this association (22).

Structurally, increased fat deposition and the heightened activation of the gland might lead to increased adrenal volume (23). This increase in cortisol and aldosterone further exacerbates weight gain and metabolic health, creating a vicious cycle of obesity. Additionally, excessive production of adrenal androgens has also been noted, which may disrupt normal growth and pubertal development (22).

### Pancreas

One of the major hormones secreted by the pancreas is glucagon. Few studies are reported in the literature regarding alterations in glucagon levels in the pediatric population with obesity. However, a study showed that patients with obesity and insulin resistance had higher fasting glucagon levels compared to those with normal weight and those with obesity but with normal insulin sensitivity (24). Usually, glucagon levels are suppressed in response to higher glucose levels, and this effect is mainly noticed during an oral glucose tolerance test (OGTT). However, it was demonstrated that glucagon levels were elevated and minimally suppressed in those with obesity and insulin resistance during an OGTT compared to the normal weight group (24, 25). The glucagon level was also inadequately suppressed in children and adolescents with obesity during an euglycemic-hyperinsulinemic state, which usually tests for insulin tissue sensitivity with normal blood glucose levels (24). Based on all these findings, it has been suggested that alpha cells of the pancreas might have insulin resistance that prevents the effect of insulin on suppressing glucagon levels (25). Furthermore, elevations of glucagon levels have also been associated with declining glucose tolerance, hyperinsulinemia, visceral adiposity, high plasma-free fatty acids, and high plasma triglycerides (24, 25). These increased levels are also related to worsened insulin sensitivity and a decline in pancreatic beta cell function, indicating that increasing glucagon levels due to obesity are highly linked to insulin resistance in these subjects (24).

Insulin, another significant hormone secreted by the beta cells of the pancreas that its function is also affected by obesity in children and adolescents. In the pediatric population, insulin resistance is highly correlated with obesity, with nearly 38.7% of this population having insulin resistance (26). Insulin resistance, by definition, is the decreased sensitivity of tissues such as adipose, liver, and skeletal muscles to insulin levels (26). Obesity is one of the most influential factors on insulin resistance in addition to other environmental factors (26). The pathophysiology of insulin resistance in obesity depends on several factors, including lipid metabolism, which is very important. Typically, insulin reduces the release of free fatty acids from adipocytes; however, in obesity, where there is excess accumulation of free fatty acids in adipose and non-adipose tissues, insulin’s effect is attenuated, and insulin sensitivity is weakened (26, 27). The increase in serum free fatty acids levels and related metabolites accumulating in nonadipocyte tissues inhibits the insulin signaling pathway, leading to higher insulin resistance (26, 28). The increase in insulin resistance leads to a rise in insulin levels, causing a state of hyperinsulinemia in youth with obesity (26). Moreover, individuals with obesity can develop a state called glucolipotoxicity, which unfavorably affects the pathway of insulin synthesis and secretion. Glucolipotoxicity is a term that refers to the combined and harmful effects of excess glucose and free fatty acids on pancreatic beta-cell function and survival, particularly in the context of obesity and insulin resistance. Prolonged glucolipotoxicity further exacerbates insulin resistance and ultimately leads to the destruction of the pancreatic beta cells. Therefore, glucolipotoxicity is considered the most significant factor that contributes to T2D development in the childhood population (26, 29–31). Another important issue that influences insulin sensitivity is body fat distribution. Research indicates that the location of body fat—precisely, whether fat is stored peripherally or viscerally plays a crucial role in metabolic health. Lean people with fat located in the peripherals are characterized by a higher insulin sensitivity than slim individuals with intra-abdominal fat storage. Central fat distribution is linked to an increased risk of low-grade inflammation and the release of free fatty acids into the bloodstream (32). Then, again, it can negatively impact insulin signaling, making it more difficult for the body to use insulin effectively. All those factors raise the risk of childhood T2D, which was previously linked mainly to adults. However, T2D incidence has been rising extensively recently in the pediatric population, with the majority of cases affecting youth with obesity. These subjects remain at high risk of having diabetes in adulthood (26, 32).

### Adipose tissue

Adipose tissue is considered an endocrinological gland that secretes adipokines, which are signaling proteins that play critical roles in regulating various physiological processes, including metabolism, inflammation, and insulin sensitivity. Some adipokines secreted by the adipose tissues, such as leptin, adiponectin, resistin, tumor necrosis factor-alpha (TNF-α), interleukin 6 (IL-6), visfatin, omentin, chemerin, apelin, and C1q/tumor necrosis factor-related protein 1 (CTRP1) are shown to be affected by obesity in pediatric populations (33). One of the most significant adipokines released from adipose tissue is leptin. This peptide hormone is encoded by the leptin gene (LEP), also known as the obese gene (OB), located on chromosome 7 in humans. Leptin is key in regulating homeostasis energy, metabolism, and appetite. Leptin’s prime place of action is the central nervous system, in the brainstem and hypothalamus. It is a main regulator of satiety, promoting lipolysis and increasing free fatty acid levels (26, 34). Leptin and insulin are found to regulate each other where insulin induces the release of leptin, whereas leptin, in return, leads to a decrease in insulin synthesis and secretion (26). Several studies revealed that leptin levels in children with obesity were higher than those of normal weight (33–36). Hyperleptinemia has been correlated with increased insulin resistance in these patients (26, 36). Individuals with obesity have up to four times higher leptin than lean individuals due to their increased fat mass. Then, leptin resistance is a common finding in children with obesity (37). This ultimately leads to the desensitization of brain receptors, where the brain fails to respond to leptin’s satiety signals and leads to a persistent feeling of hunger, which provokes continuous eating and weight gain, yielding a greater BMI among these individuals (37–39). Some metabolic dysfunctions, such as impaired glucose metabolism and insulin sensitivity, are also linked to leptin resistance, making it a central factor in the pathophysiology of obesity (40).

Another main adipokine released by adipose tissue is adiponectin. It has many beneficial properties, such as antidiabetic, anti-inflammatory, and insulin sensitizer (26, 33). Adiponectin promotes fatty acid oxidation and lowers triglyceride levels, which improves insulin sensitivity (33). Studies have proved that adiponectin levels in pediatric populations with obesity are lower compared to those with normal weight (26, 33). This decrease in adiponectin levels is associated with the development of metabolic syndrome, insulin resistance, and hypertension in individuals with obesity (33).

Resistin, an adipocyte-specific hormone, is crucial in regulating metabolism and is a key link between obesity, insulin resistance, and T2D. While classified as an adipokine, resistin is also highly expressed in macrophages and contributes significantly to inflammation. As a pro-inflammatory cytokine, resistin exerts its effects through TNF-α. However, the exact molecular pathways by which resistin interacts with cells and receptors remain unclear (41). Other proinflammatory cytokines, such as TNF-α and IL-6, are primarily produced by visceral adipose tissue. These cytokines are key proinflammatory cytokines that play significant roles in the development and progression of obesity and its associated metabolic complications. Both are involved in developing insulin resistance by interfering with insulin signaling by promoting the activation of inflammatory pathways that impair the function of insulin receptors. This reduces glucose uptake by cells, contributing to hyperglycemia (41). Visfatin, also known as nicotinamide phosphoribosyl transferase (NAMPT), has insulin-like effects. It can enhance glucose uptake in cells and stimulate insulin signaling, acting as a potential regulator of glucose metabolism. In obesity, visfatin levels are commonly increased, especially in visceral fat, and are associated with low-grade inflammation. Increased visfatin concentration correlates with insulin resistance and T2D. Another proinflammatory cytokine that is also elevated in obesity is chemerin. Its higher levels are positively correlated with the amount of visceral fat, and it is related to insulin resistance, T2D, and dyslipidemia (41).

Apelin plays a role in improving vascular function and increasing insulin sensitivity. In obesity, apelin levels may decrease, contributing to insulin resistance, hypertension, and other cardiovascular risk factors. At the same time, omentin is considered to have anti-inflammatory effects. However, its levels are typically decreased in individuals with obesity and metabolic disorders. Reduced omentin levels are linked to increased fat accumulation, particularly visceral fat, and are considered a marker of metabolic dysfunction in obesity (41).

Regarding CTRP1 protein, which is known to play a role in fatty acid oxidation, it was found to be increased in patients with obesity and hyperglycemia compared to those with obesity but with normal glucose levels (27, 37). Furthermore, CTRP1 was positively correlated with higher HbA1C and glucose levels in adolescents with T2D (42). The hypothesis suggests that the increase in CTRP1 levels in those with T2D could be a compensatory mechanism since a study on mice illustrated that CTRP1 infusion was associated with lowering blood glucose levels and improving insulin sensitivity (42). Further studies are needed to explore the exact mechanism that links CTRP1 to glucose levels, as limited articles regarding this topic are present today. **Table 1** summarizes the main role of selected adipokines and their concentrations in patients with obesity.

**Table 1:** The main role of selected adipokines and their concentrations in patients with obesity.

Adipokines	Main role	Levels in individuals with obesity
Leptin (21, 23, 27, 29)	Regulates appetite, decreases hunger, and promotes satiety. Increases energy expenditure. Enhances the release of gonadotropin hormones, which are crucial for starting and sustaining normal reproductive functions.	Increased*
Adiponectin (29)	Improves insulin sensitivity. Exerts anti-inflammatory effects by decreasing the production of pro-inflammatory cytokines. Activates several signaling pathways that promote anti-inflammatory responses. Improves vascular function and reduces oxidative stress, then has cardioprotective effects.	Decreased
Resistin (29)	Negatively influences insulin signaling, then enhances insulin resistance. Increases inflammation by stimulation of the production of pro-inflammatory cytokines. Contributes to various components of metabolic syndrome development, including hypertension, dyslipidemia, and abdominal obesity.	Increased
TNF-α (22, 29)	TNF-α is a pro-inflammatory cytokine. Chronically elevated levels in individuals with obesity contribute to insulin resistance and hyperinsulinemia.	Increased
IL-6 (22, 29)	Pro-inflammatory cytokine in patients with obesity is associated with insulin resistance, hypertension, and dyslipidemia.	Increased
Visfatin (29)	It is thought to play a role in the inflammatory response. It can stimulate the production of pro-inflammatory cytokines, which may contribute to chronic inflammation often seen in obesity-related conditions.	Increased
Omentin (29)	Enhances insulin sensitivity and has anti-inflammatory properties. Positively influences lipid metabolism and has beneficial cardiovascular health.	Decreased
Chemerin (29)	Pro-inflammatory cytokine in patients with obesity is associated with insulin resistance and type 2 diabetes.	Increased
Apelin (29)	Decreases appetite and food intake. Enhances energy expenditure and lipid metabolism. Improves insulin sensitivity.	Increased*
CTRP1 (21, 30)	Improves insulin sensitivity. Anti-inflammatory properties.	Increased*

TNF-α, tumor necrosis factor alpha; interleukin-6, IL-6; CTRP1, C1q/tumor necrosis factor-related protein 1.

* A paradoxical state of resistance.

### Gastrointestinal hormones

Obesity in children has a significant role in impacting gastrointestinal (GI) hormones as well. These hormones are essential in regulating appetite, energy balance, and metabolism. Appetite and satiety are controlled through neuroendocrine feedback mechanisms, for example, by stimulating the hunger and satiety centers in the hypothalamus, more specifically in the arcuate nuclei, using many different signaling pathways (38). GI hormones, including ghrelin, glucagon-like peptide-1 (GLP-1), and peptide YY (PYY), are crucial in appetite control and energy balance.

Firstly, ghrelin is mainly produced from P/D1 enteroendocrine cells found in the stomach and intestines - Where “P” stands for “Producing” and “D” stands for “Diffuse.” It can also be produced by the kidneys, testes, and the pancreas (43). This hormone, also known as “the hunger hormone,” is responsible for stimulating hunger (orexigenic) by acting on the hypothalamus (38). In normal-weighted individuals, ghrelin levels are elevated before each meal and are suppressed afterward, but this is not the case in patients with obesity as there is no postprandial decrease in ghrelin in such individuals, and over time, ghrelin resistance is observed in these patients (43). Stress might also cause ghrelin resistance, further complicating weight control in individuals with obesity (43, 44).

Subsequently, glucagon-like peptide-1 (GLP-1) is produced by the degradation of proglucagon peptide by various organ cells such as alpha pancreatic cells, intestinal L-cells, and the nucleus of the solitary tract (45). This peptide exerts its effect by enhancing insulin secretion, decreasing glucagon secretion, delaying gastric emptying, and lowering appetite through its vagal stimulation pathway, leading to increased feelings of satiety and lower glucose levels (45). Some studies have shown that in patients with obesity, GLP-1 secretion was approximately 20% less than in those with normal weight upon oral glucose intake. However, other studies showed different results (46). Therefore, recently introducing anti-obesity drugs such as GLP-1 agonists was a significant milestone in childhood obesity treatment. The positive impact of these agents on weight loss primarily stems from their ability to slow gastric emptying, which enhances feelings of fullness and reduces appetite through the hypothalamus. Additionally, research on adults has consistently shown that GLP-1 agonists have comparable effects on major adverse cardiovascular events, overall mortality, and cardiovascular-related deaths in individuals with obesity (47).

Finally, peptide YY (PYY) is an anorexigenic hormone and is released from the L-cells in the intestines in response to food intake, especially protein intake, and is also responsible for signaling satiety and inhibiting gastric emptying and gastrointestinal motility, allowing for more extended digestion and absorption of nutrients. Research has indicated that children with obesity may exhibit altered levels of PYY compared to their healthy peers. Some studies suggest that children with obesity have lower postprandial levels of PYY, which could contribute to their difficulty in regulating appetite and satiety. This reduced responsiveness may lead to increased food and calorie intake, prolonged eating sessions, and difficulty achieving or maintaining an optimal body mass (48, 49).

### Pituitary gland

Childhood obesity’s effects on the pituitary gland are also observed. For example, youth with obesity experience a decrease in the secretion of the growth hormone (GH), altered thyroid-stimulating hormone (TSH) levels, and early activation of the hypothalamic-pituitary-gonadal (HPG) axis, leading to impaired growth, insulin resistance, and early puberty (12, 50, 51).

Growth hormone (GH) is responsible for children’s bone growth, body composition, and metabolism. In patients with obesity, GH levels are remarkably decreased by almost 50% compared to healthy peers (50, 52). Elevated insulin levels and a high-fat mass cause increased somatostatin release, which is a GH inhibitor, leading to a significant decrease in GH levels (12, 52). An important feedback mechanism causing lower GH levels is the elevated insulin-like growth factor 1 (IGF-1) due to high adiposity in individuals with obesity (12, 52).

In lean people, GH promotes muscle growth and muscle development; on the other hand, those with obesity have lower GH, thus lower lean body mass (LBM) and increased fat mass (53). Concerning an individual’s height, a decrease in GH leads to premature epiphyseal closure and earlier skeletal maturation, which can ultimately cause a reduced adult height (53). Elevated leptin levels can also accelerate this process and can promote the early onset of puberty as well, especially in girls (51, 54). Another metabolic role of GH is lipolysis, the breakdown of fat into free fatty acids and glycerol. In obesity, this metabolic process is limited, and there is more fat accumulation, which in turn leads to insulin resistance and metabolic complications (52, 53).

GH therapy has been shown to increase LBM while decreasing visceral fat mass, causing an improved overall body composition in obese patients. It is important to note that GH therapy does not lead to significant weight loss; rather, it causes an improvement in body composition and insulin sensitivity, which is commonly impaired in those children (50, 52).

### Gonads

In children with obesity, the normal hormonal regulation essential for growth, pubertal milestones, and fertility is disrupted. According to a German study, the early development of pubertal milestones and the accelerated skeletal growth rate were associated with increased levels of leptin, insulin, and IGF-1 in children with obesity (55). These differences were more frequent in girls with obesity, with an occurrence rate of 48% compared to 8.73% in the normal weight group and 6.78% in boys with obesity, as opposed to 2.86% in the average weight category (54).

The link between obesity and precocious puberty could be explained through several hypotheses. Firstly, increased levels of leptin in both genders and kisspeptin in girls, both crucial for the initiation and progression of puberty, have been found in children with obesity (56). Moreover, hyperinsulinemia increases aromatase activity in adipocytes, as well as androgen production in the adrenals and ovaries, decreases sex hormone-binding globulin (SHBG) synthesis (56, 57), and thus plays a significant role in the development of pubarche (58).

In girls, the earlier development of adrenarche and thelarche is mainly affected by the increased level of estrogen, which can then further disrupt the hypothalamic-pituitary-gonadal (HPG) axis and increase the risk of polycystic ovary syndrome (PCOS), irregular menses, and infertility (56). At the same time, the increased level of androgen leads to the evolution of precocious pubic and axillary hair, mild acne, pubertal sweat odor, and moderately accelerated bone age growth (58). Additionally, the decrease in IGF-1-binding protein levels increases free IGF-1, an essential factor in the early onset of thelarche and menarche (59).

**Figure 2:**
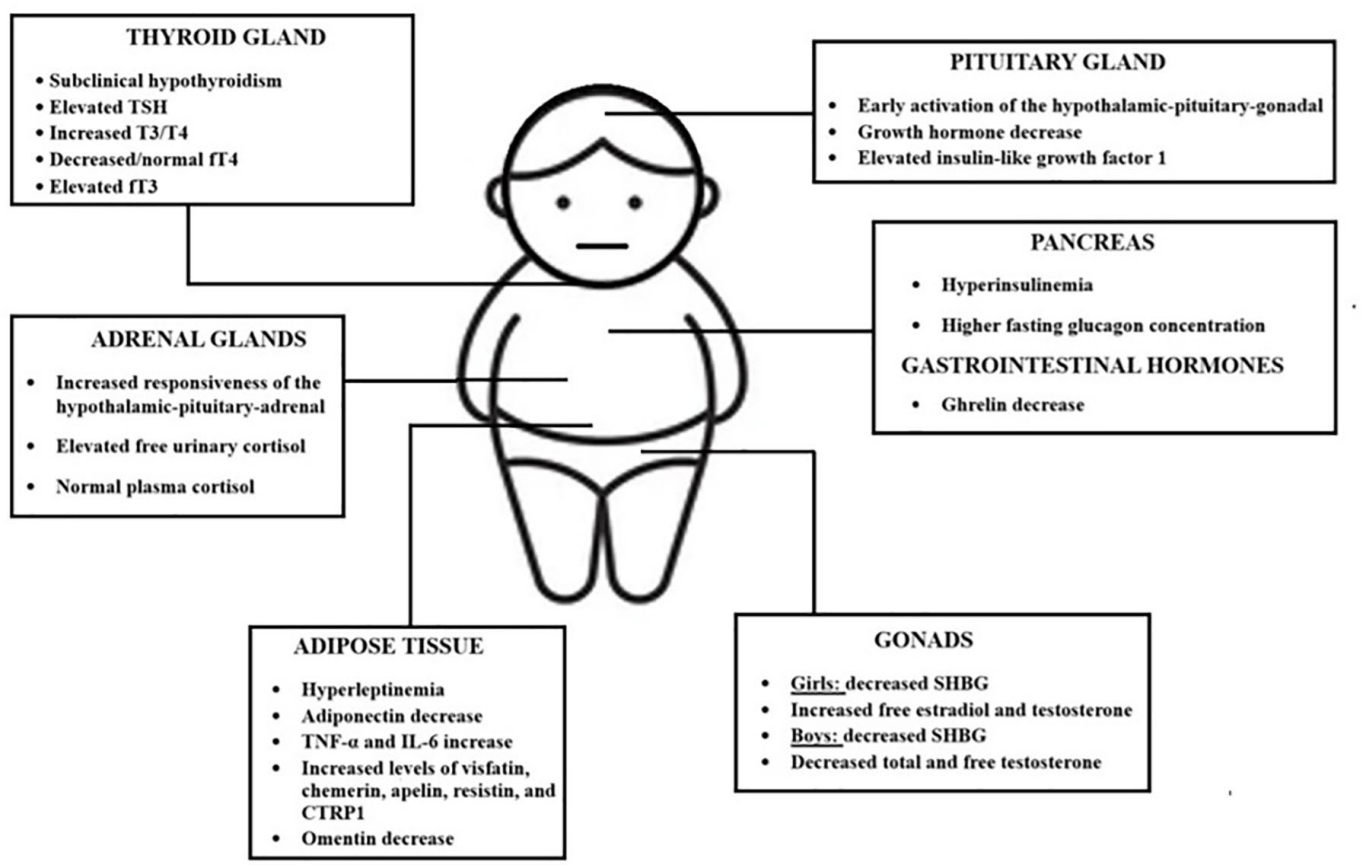
The most important childhood obesity-related endocrine changes. TSH; thyroid stimulating hormone; T3, Triiodothyronine; T4, thyroxine; fT4, free thyroxine; FT3, free triiodothyronine; TNF-α, tumor necrosis factor alpha; IL-6, interleukin-6; CTRP1, C1q/tumor necrosis factor-related protein 1.

In boys, high levels of estrogen suppress the production of testosterone, causing gynecomastia, as well as an impairment in spermatogenesis (60). Levels of IGF-1 are high in the pre-pubertal state and are associated with earlier gonadarche and increased height (59). However, it has been shown that during puberty, levels of testosterone and IGF-1 decreased in boys, exhibiting a decline in growth rate (55). The effect of obesity on puberty in boys is inconclusive, and the exact mechanism of the decrease in IGF-1 levels during puberty is still unclear, however it is said to be related to the drop in testosterone (55). **Figure 2** summarizes the most obesity-related endocrine changes in childhood populations.

## Conclusions

In conclusion, childhood obesity presents a multifaceted challenge to endocrine health, with significant implications for both immediate and long-term well-being. The endocrine dysregulation observed in children with obesity, including insulin resistance, thyroid dysfunction, altered reproductive development, and hormonal imbalances like leptin resistance and cortisol dysregulation, underscores the urgent need for targeted interventions. The complex relationship between obesity and endocrine pathways requires a holistic approach to prevention, early detection, and treatment. A comprehensive public health strategy that addresses both the physiological and hormonal aspects of childhood obesity is essential to mitigate its profound consequences and reduce the risk of chronic diseases, such as T2D and cardiovascular complications, in affected children. Understanding these interconnections is crucial for crafting effective policies and clinical approaches to improve the health of this vulnerable population.
